# Brillouin Scattering Selection Rules in Polarization-Sensitive Photonic Resonators

**DOI:** 10.1021/acsphotonics.3c00186

**Published:** 2023-06-01

**Authors:** Anne Rodriguez, Priya Priya, Edson R. Cardozo de Oliveira, Abdelmounaim Harouri, Isabelle Sagnes, Florian Pastier, Luc Le Gratiet, Martina Morassi, Aristide Lemaître, Loïc Lanco, Martin Esmann, Norberto Daniel Lanzillotti-Kimura

**Affiliations:** # Université Paris-Saclay, CNRS, Centre de Nanosciences et de Nanotechnologies, 10 Boulevard Thomas Gobert, 91120 Palaiseau, France; ‡ Quandela SAS, 10 Boulevard Thomas Gobert, 91120 Palaiseau, France; § Université Paris-Cité, CNRS, Centre de Nanosciences et de Nanotechnologies, 10 Boulevard Thomas Gobert, 91120 Palaiseau, France

**Keywords:** Brillouin scattering, Polarization, Elliptical micropillar resonators

## Abstract

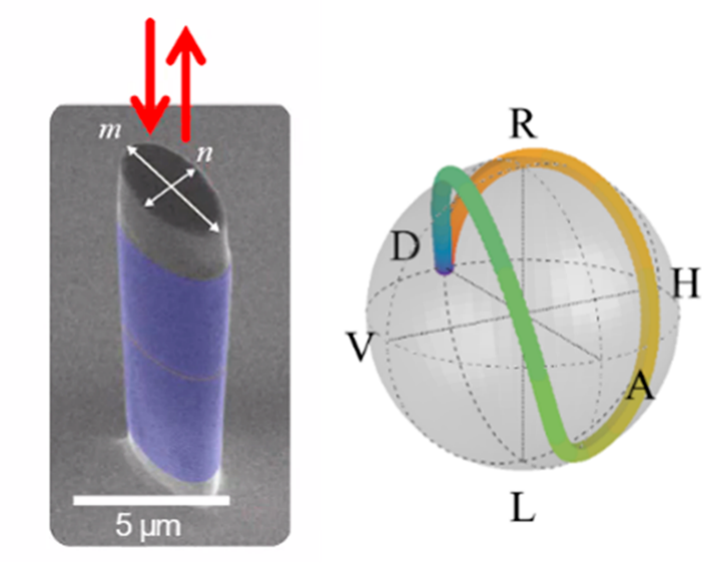

Spontaneous Brillouin scattering in bulk crystalline solids is governed by the intrinsic selection rules locking the relative polarization of the excitation laser and the Brillouin signal. In this work, we independently manipulate the polarization of the two by employing polarization-sensitive optical resonances in elliptical micropillars to induce a wavelength-dependent rotation of the polarization states. Consequently, a polarization-based filtering technique allows us to measure acoustic phonons with frequencies difficult to access with standard Brillouin and Raman spectroscopies. This technique can be extended to other polarization-sensitive optical systems, such as plasmonic, photonic, or birefringent nanostructures, and finds applications in optomechanical, optoelectronic, and quantum optics devices.

## Introduction

Brillouin scattering, the inelastic scattering of light with acoustic phonons, is extensively used in material characterization, biological imaging, and optical and optoelectronic devices.^1^ In Brillouin scattering processes, the selection rules formally constrain energy, direction, and polarization of the scattered photons for a given input state. These selection rules in crystalline solids are usually taken as intrinsic material properties, locking the relative polarization of excitation and signal states.^2^ For example, exciting a zinc blende material, such as GaAs, along the [001] direction, the backscattered Brillouin signal preserves the polarization state of the excitation laser source. Using artificial birefringent microstructures, these selection rules can be broken.

It has been shown that the wave vector selection rules of spontaneous Brillouin scattering can be modified in microstructures.^3^ Also, the scattering cross sections can be largely enhanced by means of micro- and nanostructures, such as microcavities^3,4^ and surfaces.^5,6^ More recently, polarization control in stimulated Brillouin scattering has been reported in birefringent photonic crystal fibers, polarization maintaining fibers, and nanofibers.^7−9^ It is intriguing to explore the control of polarization in the spontaneous Brillouin scattering regime in microstructures which is otherwise subtle to observe in fibers. In this work, we show that not only the wave vector selection rules and scattering cross sections but also the polarization selection rules of spontaneous Brillouin scattering can be strongly modified in polarization-sensitive photonic resonators, such as elliptical micropillars,^10,11^ optical nanoantennas,^12−14^ and metasurfaces.^15,16^

Here, we introduce elliptical optical micropillar resonators to control Brillouin scattering polarization selection rules. Due to the anisotropy of the micropillar cross-section, it features two confined optical cavity eigenmodes with orthogonal linear polarizations^11,17,18^ and nondegenerate energies. The energy separation of the modes can be controlled by the size and ellipticity of the pillar. The two orthogonal resonances can induce an energy-dependent polarization rotation of light. This property of elliptical micropillars has already been employed for polarization-dependent emission of quantum-dot based single-photon sources.^10,19−22^

Since polarization rotation is strongly wavelength-dependent, the inelastically scattered Brillouin emission undergoes a different rotation of polarization than the incident excitation laser. By properly choosing the polarization and wavelength of the excitation laser, the Brillouin signal and the reflected excitation laser may emerge in orthogonal polarization states enabling an efficient cross-polarization detection scheme with largely suppressed background from the excitation laser. This overcomes one of the critical challenges in Brillouin spectroscopy, which is to detect the inelastically scattered photons while rejecting the reflected part of the excitation laser source (typically 5–6 orders of magnitude more powerful).

## Experimental Methods

**Figure 1:**
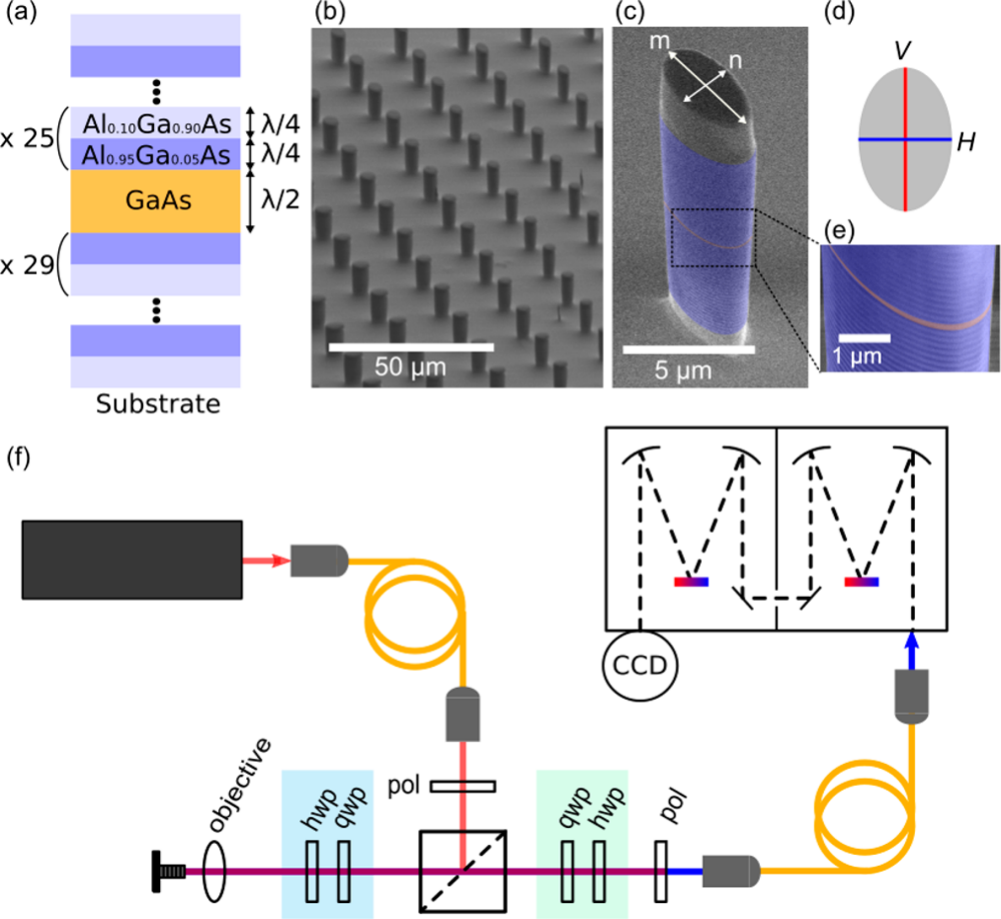
Micropillar resonator and experimental setup. (a) Schematic of the vertical layer structure of the micropillar with two distributed Bragg reflectors (DBRs) enclosing a resonant spacer. (b) Scanning electron microscope (SEM) image of an array of micropillars with various sizes and ellipticities. (c) SEM image of an elliptical pillar, *m* and *n* are the major and minor axis lengths of the cross-section, respectively. The vertical structure consists of two GaAs/AlAs DBRs (blue) enclosing a resonant half-wavelength GaAs spacer layer (orange). (d) Schematic of the pillar cross-section. The two fundamental optical eigenmodes are of orthogonal linear polarizations *V* and *H*, polarized along the two axes of the cross-section. (e) Zoom-in on the structure of a pillar. (f) Experimental scheme. The polarized excitation laser is focused to a spot of 2.2 μm diameter on the sample with an objective lens of 0.7 NA. The Brillouin signal is collected through a single mode fiber before entering a double spectrometer in additive mode. Pol stands for polarizer, and qwp (hwp) stands for quarter- (half-) waveplate.

The sample under study is grown on a (001)-oriented GaAs substrate by molecular-beam epitaxy. It consists of an acousto-optical microcavity with two distributed Bragg reflectors (DBRs) enclosing a resonant spacer^28,29^ with an optical path length of λ/2 at a resonance wavelength of around λ ∼ 900 nm in vacuum. The top (bottom) optical DBR is formed by 25 (29) periods of Ga_0.9_Al_0.1_As/Ga_0.05_Al_0.95_As bilayers (λ/4/λ/4) (Figure 1(a)). From this planar acousto-optical cavity, micropillars of various sizes and ellipticities are fabricated by optical lithography followed by inductively coupled plasma etching (Figure 1(b)). Figure 1(c) shows a scanning electron microscope (SEM) image of an individual micropillar where the DBRs are highlighted in blue and the spacer in orange. A schematic of the cross-section and a zoom on the multilayered structure are shown in Figure 1(d) and Figure 1(e), respectively. The micropillars confine an optical mode with typical Q-factors of 11 000. Corresponding field distributions were previously calculated in ref (22). This structure simultaneously confines acoustic phonons at around 18 GHz, which translates into equal wavelengths of the confined optical and acoustic modes with perfect colocalization.^23−25^

Figure 1(f) shows a schematic of the optical spectroscopy setup implemented using polarization as a Brillouin filtering technique in a backscattering configuration. A collimated laser beam from a tunable continuous wave (cw) Ti:sapphire laser (M2 SolsTis) is used as an excitation light source. The incident beam polarization is initialized with a polarizer and a set of a quarter- and a half-waveplate (qwp and hwp). The incident laser beam is focused on the sample with a spot diameter of approximately 2.2 μm using a NA = 0.7 objective lens. The reflected signal is collected through the same objective and waveplates. A second set of waveplates in the collection path allows us to choose the polarization basis for the collection, while a second polarizer acts as an analyzer. The transmission of the polarizer in the collection path is 86% at the wavelength of interest around 900 nm. The signal is collected using a single-mode fiber acting as a spatial filter, and finally analyzed with a double monochromator operating in additive mode (Jobin Yvon HRD 2) and a charged-coupled device (CCD, LN 100BR Detector Excelon Princeton instruments). The typical integration time of the CCD detector is 0.1 s.

**Figure 2:**
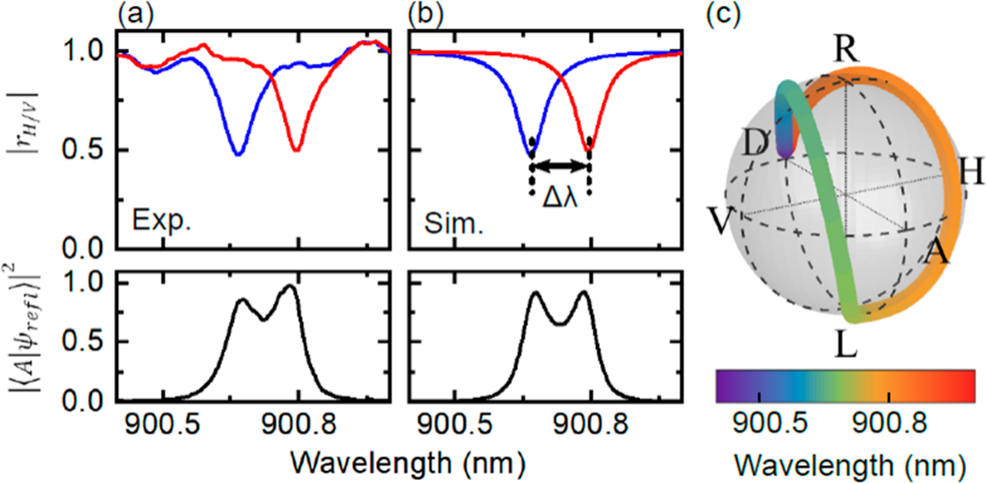
Polarization rotation by an elliptical micropillar resonator. (a) Experimental reflectivity spectra of an elliptical micropillar of ellipticity *e* = 0.41 with *m* = 4 μm. The blue (red) spectrum is measured with a linear polarization aligned with the minor (major) axis of the elliptical pillar cross-section. Two clear optical modes are observed at different central wavelengths. Bottom panel: Spectrum of the reflected excitation laser with rotation of polarization. The incident laser is polarized along *D*, while the detection projects along *A*. (b) Calculated reflectivity spectra of the two optical modes presented in panel (a). Δλ = 0.127 nm is the splitting between the two linearly polarized eigenmodes. Bottom panel: Calculated spectrum of the reflected laser with rotation of polarization. (c) Poincaré sphere displaying the calculated wavelength-dependent polarization state |*ψ*_refl_⟩ of the reflected excitation laser.

For the measurements of polarization-dependent reflectivity and polarization rotation in Figure 2, we set the incident beam polarization (|*ψ*_in_⟩) to diagonal . The reflectivity was analyzed in the *H/V* basis. To measure the polarization rotation, the collection was performed in the antidiagonal state *A*.

**Figure 3:**
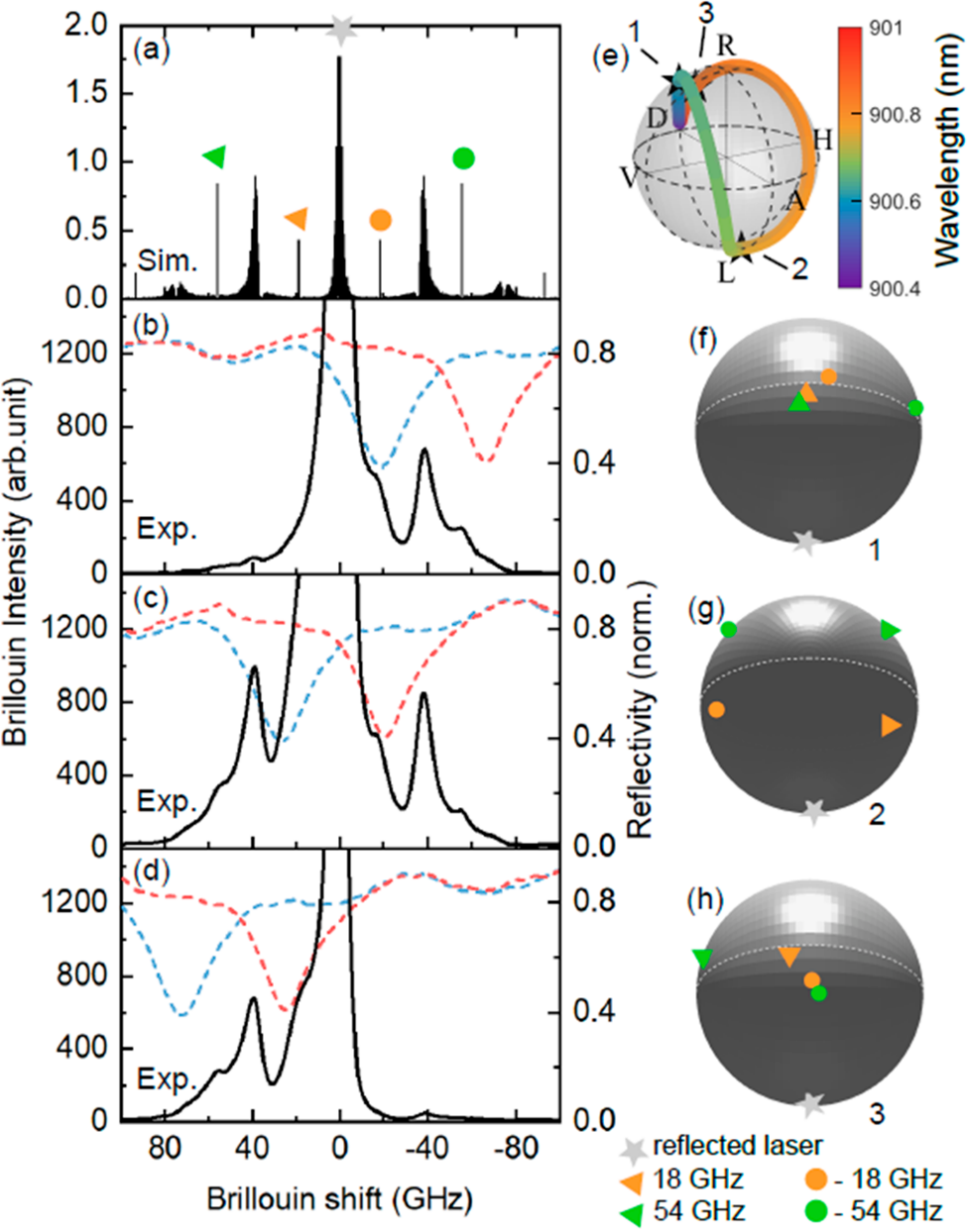
Polarization rotation-enabled optical filtering. (a) Simulated Brillouin spectrum for a planar cavity with the multilayer structure shown in Figure 1(a). Experimental Brillouin spectra acquired with the excitation laser blue-detuned from the optical modes (b), between the optical modes (c), and red-detuned from the optical modes (d). The peak at ±18 GHz corresponds to the fundamental confined acoustic mode of the resonator. The peak at ∼±37 GHz corresponds to bulk Brillouin scattering from the substrate. The peak at ±54 GHz corresponds to the third harmonic of the confined mode. (e) Simulated polarization states of the reflected laser for the wavelengths used in panels (b–d) indicated with stars on the Poincaré sphere. (f, g, h) Simulated polarization of the reflected excitation laser and Brillouin signals ((b, c, d), respectively) plotted on Poincaré spheres rotated such that the reflected laser state is localized at the South pole. The excursion of the Brillouin polarization states from the South pole is a direct indication of strongly modified polarization selection rules enabling a polarization filtering protocol.

For the Brillouin spectra presented in Figure 3, the incident excitation laser power was set to 50 μW. To measure the Brillouin signal, the collection was performed in the state orthogonal with respect to the reflected excitation laser |*ψ*_refl_⟩ (eq 3). The extinction ratio of the reflected excitation laser provided by the polarization filtering is 45:1 for Figure 3(c) and 78:1 for panels 3(b) and 3(d). These extinction ratios were measured before the collection fiber. The use of a single mode fiber further increases the purity of the collected Brillouin signal, since diffusely scattered and diffracted contributions of the returning excitation laser are rejected through spatial filtering.^26^ See Supporting Information section I for further experimental details.

The simulation of the Brillouin spectrum presented in Figure 3(a) assumes a purely photoelastic interaction.^27^ For the numerical implementation, we use a transfer matrix method with nominal material properties and assume a planar structure. The spectrum was convoluted with a Gaussian of 0.025 GHz full width at half-maximum.

**Figure 4:**
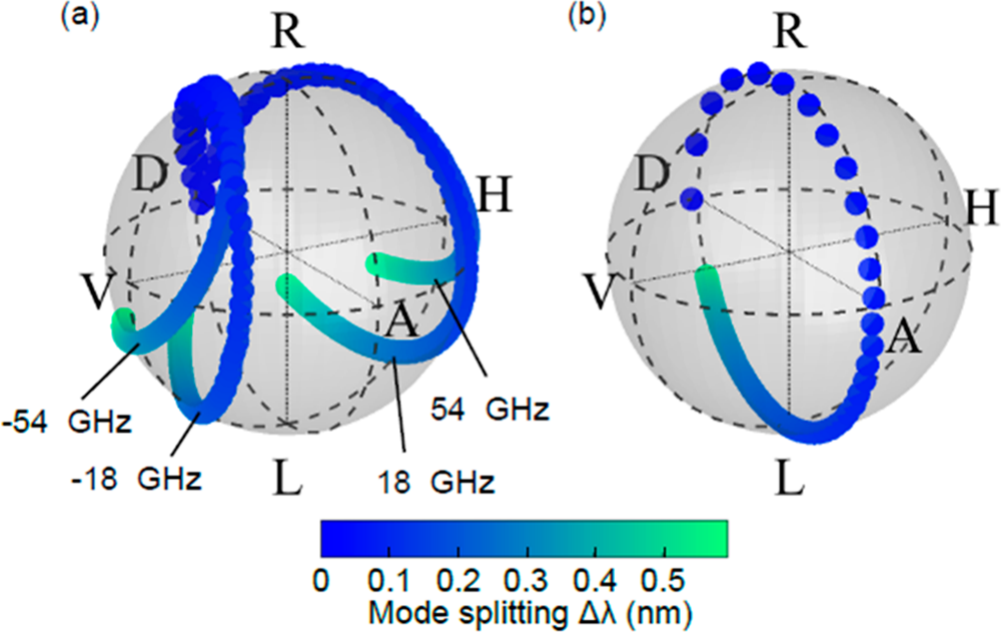
Effect of the micropillar ellipticity on the scattering selection rules. (a) Brillouin polarization states for the fundamental and third harmonic of the confined acoustic mode as a function of the mode splitting Δλ between the two optical modes in elliptical micropillars. The considered case is the particular configuration of an excitation laser with diagonal polarization and tuned between the two optical modes. For zero separation, the Brillouin polarization states coincide with the excitation laser. By increasing the separation, the modes describe frequency-dependent trajectories that span the full Poincaré sphere. (b) Reflected excitation laser polarization state as a function of the separation between the two optical modes in elliptical micropillars with same excitation conditions as in panel (a). The described trajectory evolves along a meridian of the Poincaré sphere. The difference in polarization states observed between panels (a) and (b) enables an efficient polarization filtering protocol.

The polarization-dependent studies are based on the Jones matrices formalism (see Supporting Information section II). First, we fit the reflectivity contrast, resonance wavelength and line width of the polarization dependent eigenmodes of the elliptical micropillar using a Lorentzian model (Figure 2(b), top). The resulting parameters are then used as input for the Jones matrices to compute the simulated results shown in Figure 2(b), bottom, Figure 2(c), and Figure 3(e–h). The same line width and reflectivity contrasts were used as inputs for the additional simulation results presented in Figure 4.

## Results and Discussion

An elliptical micropillar cavity has two fundamental optical eigenmodes |*H*⟩ and |*V*⟩ of orthogonal linear polarizations (horizontal/vertical, *H/V*). They are polarized along the minor/major axis of the elliptical cross-section, as shown in Figure 1(c), and have nondegenerate resonance frequencies *ω*_c,H_ and *ω*_c,V_, respectively. In what follows, we establish the polarization states of the reflected laser and the Brillouin signal in this system. Considering an incoming excitation laser field of frequency *ω*_in_, the input polarization state is
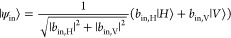
1

The associated intracavity field takes the form:
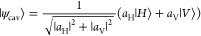
2and the resulting reflected excitation laser field is
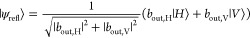
3

In eqs 1–3, *b*_in,H/V_, *b*_out,H/V_, and *a*_H/V_ are the polarization amplitudes of the incoming, reflected and intracavity excitation laser fields, respectively. In eq 2,  with κ the cavity damping rate.^30,31^

The reflected fields in eq 3 are obtained using the standard input–output equations , with κ_t_ representing the polarization-dependent top DBR leakage rate. *b*_out_ represents the reflected field as an interference between the input light directly reflected by the top DBR and the light emerging from the cavity.

The Brillouin scattering field inside the GaAs cavity spacer has the same polarization amplitudes as the intracavity polarization state |*ψ*_cav_⟩ of the excitation laser but at a different frequency *ω*_B_. The polarization state of Brillouin scattering |*ψ*_B_⟩ outside the cavity is then given by
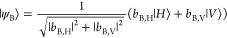
4where . Here, *b*_B,H/V_ is proportional to the product of two terms: the first term depends on the frequency of the Brillouin signal, the second term depends on the incoming excitation laser frequency and polarization through the intracavity amplitude *a*_H/V_. Consequently, the polarization state of the Brillouin signal can be controlled by the micropillar geometry and is not only dictated by the material-dependent polarization selection rules. Moreover, the reflected laser and the Brillouin signal polarization states experience different degrees of polarization rotation and can hence be discriminated by polarization filtering.

For the experimental demonstration of these phenomena, we study a sample consisting of arrays of GaAlAs micropillars with various sizes and ellipticities (Figure 1(a)). Vertically, the pillars consist of two (λ/4/λ/4) GaAs/AlAs DBRs enclosing a resonant λ/2 GaAs spacer layer. They act as an optical resonator for near-infrared photons and as an acoustic resonator for longitudinal acoustic phonons around 18 GHz.^23,24,32,33^ The micropillar ellipticity is , where *m* and *n* are the major and minor axis length of the elliptical cross-section,^11^ see Figure 1(b, c).

Figure 1(f) presents the experimental spectroscopy setup to measure polarization-resolved optical reflectivity and Brillouin scattering. The polarizations of the incident excitation laser and collected signal can be controlled independently.

We measure the polarization-dependent optical reflectivity (Figure 2) for an elliptical micropillar of ellipticity *e* = 0.41 with *m* = 4 μm. We prepare an incident excitation laser with diagonal polarization  and detect two projections |⟨*ψ*_det_|*ψ*_refl_⟩|^2^ with |*ψ*_det_⟩ = |*H*⟩, |*V*⟩. By scanning the laser frequency, two well-defined polarization-dependent optical modes emerge (panel (a), top). The blue (red) mode corresponds to *H(V)* polarization, associated with the minor (major) axis of the pillar cross-section, respectively. Using the definitions in eqs 1–3, the polarization-dependent complex reflection coefficients *r*_V_ and *r*_H_ are
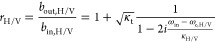
5

The cavity damping κ_(H,V)_ includes sidewall losses and leakage through the top and bottom DBRs.^31^ In Figure 2(a) (bottom), we show the antidiagonal (|*A*⟩) component of the reflected signal, i.e., |*ψ*_det_⟩ = |*A*⟩ collecting light in a cross-polarization scheme. Effectively, this signal represents excitation laser photons, whose linear polarization state has been rotated from |*D*⟩ to |*A*⟩ upon reflection. The measured spectrum exhibits two maxima that are roughly localized at the spectral positions of the eigenmodes of the elliptical micropillar. Outside the resonances, the rotated signal is practically zero. Figure 2(b) shows a simulation of these rotated signals using the Jones matrices formalism (see Supporting Information section II) resulting in excellent agreement with the experimental spectrum.^34,35^ To convey the full information of the reflected signals, we in addition plot the simulated polarization state of the reflected excitation laser on a Poincaré sphere in panel (c). As a function of wavelength, the reflected state undergoes a trajectory that spans the full sphere.

In what follows, we exploit the difference in rotation of polarization between the reflected excitation laser field and the much weaker Brillouin scattered component for a polarization filtering protocol. The difference in rotation of polarization implies |*ψ*_in_⟩ ≠ |*ψ*_refl_⟩ ≠ |*ψ*_B_⟩. That is, the backscattering Brillouin selection rule in bulk GaAs (|*ψ*_in_⟩ = |*ψ*_refl_⟩ = |*ψ*_B_⟩ for excitation along the [001] direction) is altered by engineering the optical modes in a cavity. The filtering is then achieved by detecting the Brillouin signal in a cross-polarization geometry ensuring that ⟨*ψ*_det_|*ψ*_refl_⟩ = 0. That is
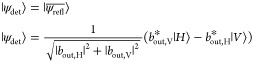
6

Note that here the cross-polarization condition depends on the excitation laser wavelength due to the wavelength-dependence of |*ψ*_refl_⟩ as shown in Figure 2.

Figure 3(a) shows a simulated Brillouin spectrum of the vertical microcavity structure (see Figure 1(a)) which illustrates the frequency offset between the Brillouin signal and the excitation laser (See Supporting Information section III). Positive (+) and negative (−) Brillouin shifts mark the anti-Stokes and Stokes signal, respectively. Peaks at ±18 GHz, ±54 GHz, and ±90 GHz correspond to the Stokes and anti-Stokes of the fundamental acoustic mode confined in the cavity and its odd harmonics.^27,32,36^ In contrast, the peaks at ∼±37 GHz correspond to bulk Brillouin scattering from the GaAs substrate. Panels (b–d) display experimental Brillouin spectra measured at excitation laser wavelengths of 900.615 nm, 900.737 nm, and 900.860 nm, respectively. For reference, the polarization-dependent optical reflectivity is included in each panel with dashed lines. Panel 3(e) shows the same wavelength-dependent trace of the reflected excitation laser plotted in Figure 2(c) but with numbered black stars indicating the polarization state of the reflected excitation laser for the cases measured in panels (b)–(d). Panels (f)–(h) show the simulated polarization states of the reflected excitation laser, |*ψ*_refl_⟩, and the Brillouin scattered signals, |*ψ*_B_⟩, on the same Poincaré sphere for each of the three measured cases. The polarization states are simulated using Jones matrices and considering Brillouin scattering as a source term inside the cavity spacer. For clarity reasons, the Poincaré spheres are oriented such that |*ψ*_refl_⟩ is always located at the South pole of the sphere, while |*ψ*_det_⟩ is located at the opposing North pole.

Figure 3(b) displays a Brillouin spectrum obtained with the excitation laser blue-detuned from the cavity modes, at a wavelength of 900.615 nm. The observed Stokes Brillouin spectrum shows that the fundamental mode at −18 GHz is resonant with the *H*-polarized cavity mode, and the third harmonic, at −54 GHz, is coupled to the *V*-polarized cavity mode. The anti-Stokes modes are off-resonant with respect to both cavity modes and hence appear with much lower intensity in the measurement. Panel (f) shows that the four relevant signals experience a rotation of polarization that makes them detectable in the cross-polarization scheme, since none of them coincides with the South pole, i.e., they all contain at least a partial polarization component orthogonal to the reflected excitation laser. While the amplitude of the polarization component orthogonal to the reflected excitation laser is similar for all four Brillouin components, only the Stokes components are enhanced by the presence of the optical cavity modes.

Figure 3(c) shows a Brillouin spectrum measured with the excitation laser tuned between the optical cavity modes, at a wavelength of 900.737 nm. As shown on the Poincaré sphere (panel (g)), both Stokes and anti-Stokes scattering modes at ±18 and ±54 GHz have polarizations away from the South pole, with the modes at ±54 GHz closer to the North pole, implying a better polarization filtering condition for these modes. In contrast to panel (b), both the Stokes and anti-Stokes modes are enhanced by the coupling to the optical cavity modes.

Finally, when red-detuning the excitation laser, the measurement shows selective enhancement of the anti-Stokes components, which are tuned in resonance with the optical micropillar modes. Figure 3(d) shows a corresponding spectrum obtained with the excitation laser at 900.860 nm. The observed anti-Stokes Brillouin spectrum exhibits the mode at +18 GHz, which is coupled to the *V*-polarized optical cavity, and the mode at +54 GHz coupled to the *H*-polarized cavity mode. The polarization states on the Poincaré sphere (panel (h)) are mirror images of the states in panel (f), i.e., again all the Brillouin components of interest are away from the South pole.

These measurements clearly illustrate that different bands of the Brillouin spectrum can be selectively measured in an elliptical micropillar by properly choosing the wavelength and polarization state of the incident excitation laser. The configuration for cross-polarized filtering would become optimal for the reflected excitation laser and the Brillouin signal in orthogonal polarization states, i.e., , such that the Brillouin signal is fully transmitted by the analyzing polarizer.

So far, we have solely discussed the excitation conditions as a means to optimize the measurement efficiency. In Figure 4, we theoretically analyze how changing the ellipticity of the micropillar further alters the scattering selection rules. Figure 4(a) shows the calculated polarization state of the Brillouin signal as a function of the micropillar ellipticity while keeping the excitation laser wavelength centered between the two optical cavity modes and the input polarization along *D*. The trajectory of the Brillouin signal shows that the relation between the incident excitation laser and the signal polarization states is strongly modified, again breaking the intrinsic selection rules. Figure 4(b) shows the reflected excitation laser polarization state under the same excitation conditions. The resulting state is always different from the corresponding Brillouin states, i.e., ⟨*ψ*_refl_|*ψ*_B_⟩ ≠ 1, meaning that cross-polarized detection is always possible for nondegenerate polarization-dependent optical modes. The accessible phonon frequency band is determined by the separation of the optical cavity modes. In elliptical micropillars this separation typically reaches up to 10 meV (2.5 THz).^10,11^

## Conclusion and Outlook

We have theoretically proposed and experimentally demonstrated a strategy to independently manipulate the polarization state of a Brillouin scattered signal and the excitation laser. This strong modification of the polarization selection rules is achieved in elliptical micropillar cavities presenting polarization-sensitive optical cavity modes. A similar behavior was observed in birefregent and gyrotropic materials.^37,38^ Due to the wavelength-dependent birefringence of the elliptical pillar, the reflected excitation laser beam and the Brillouin scattered signal encounter different rotations of polarization, enabling a cross-polarized filtering scheme. Using the excitation laser wavelength as a tuning parameter in the measurement, different frequency bands in the Stokes and anti-Stokes Brillouin scattering spectra are selectively accessible. In the studied case, the cavity mode lifetimes, spectral separation determined via the ellipticity, and excitation laser wavelength and polarization define the parameter space to maximize cavity-enhanced signals under optimal polarization-filtering conditions. The same working principle applies to any photonic system with localized, polarization-sensitive modes, such as plasmonic resonators, photonic crystals, and birefringent micro- and nanostructures. This phenomenon can also be observed for inelastic light scattering by other excitations such as magnons, provided that the polarization state of the scattered excitation laser can be defined on the Poincaré sphere.

The presented technique is particularly important for studying phonons with frequencies between 1 GHz and 1 THz, which are relevant for thermal transport and telecommunication applications. In this range, standard Raman spectroscopy techniques lack enough resolution and standard Brillouin scattering techniques lack versatility in tuning the excitation laser wavelength.^26,39^ The polarization control protocol presented here will thus find applications in the engineering of light–matter interactions in optomechanical, optoelectronic, and quantum optics devices.^10,40,41^

## Supporting Information Available

Experimental details, Jones matrices formalism, and optical and acoustic fields (PDF)
