# Supplementary material for: Brillouin Scattering Selection Rules in Polarization-Sensitive Photonic Resonators
Source: ACS Photonics. 2023 Jun 1;10(6):1687–93. doi: 10.1021/acsphotonics.3c00186 (PMC10289090; doi:10.1021/acsphotonics.3c00186)
Supplement: Supplementary file 1 — ph3c00186_si_001.pdf [file ph3c00186_si_001.pdf]

Supporting information for:

Brillouin scattering selection rules in polarization-sensitive photonic resonators

Anne Rodriguez,[1] Priya Priya,[1] Edson R. Cardozo de Oliveira,[1] Abdelmounaim Harouri,[1] Isabelle Sagnes,[1] Florian Pastier,[2] Luc Le Gratiet,[1] Martina Morassi,[1] Aristide Lemaître,[1] Loïc Lanco,[3] Martin Esmann,[1,†] and Norberto Daniel Lanzillotti-Kimura,[1,*]

1. Université Paris-Saclay, CNRS, Centre de Nanosciences et de Nanotechnologies, 10 Boulevard Thomas Gobert, 91120 Palaiseau, France
2. Quandela SAS, 10 Boulevard Thomas Gobert, 91120 Palaiseau, France
3. Université Paris-Cité, CNRS, Centre de Nanosciences et de Nanotechnologies, 10 Boulevard Thomas Gobert, 91120 Palaiseau, France
[†] present address: Institute for Physics, Carl von Ossietzky University, 26129 Oldenburg, Germany

I. Experimental details

The experimental setup (see Fig. 1(f) of the main text) contains the following optical components:

Waveplates: Foctek achromatic waveplates working in the 700 – 1000 nm range.

Polarizers: Codixx with transmittance >91% in the 900 – 1200 nm range (IR 1100 BC4 CW02).

Collection fiber: Thorlabs single mode fiber 650 – 1050 nm range (P4-780AR).

Collimators: Thorlabs zoom fiber collimator 650 – 1050 nm range (ZC618APC-B).

Double monochromator: Jobin Yvon HRD 2

CCD camera: LN 100BR Detector Excelon Princeton instruments

II. Jones Formalism

The Jones formalism is employed to describe the polarization state of a plane wave and its evolution through an optical system. In this formalism, each optical element is represented by a Jones matrix (see Table 1) in the *H/V* polarization basis. The polarization of the incident beam before the sample is controlled by θ_1 and θ_2, the angles of the quarter and half waveplates with their fast axis, respectively (see Fig. 1(f) of the main text for a detailed sketch of the experimental setup). The rotation of the waveplates with respect to the H/V basis is then captured using the following additional rotation matrix:

$$M = \begin{bmatrix} \cos\theta & -\sin\theta \\ \sin\theta & \cos\theta \end{bmatrix}.$$

The Jones matrix of the sample is given by:

$$M_{sample} = \begin{bmatrix} r_H & 0 \\ 0 & r_V \end{bmatrix},$$

where $r_{H/V}$ are the reflectivity coefficients defined in Equation (5) of the paper. The reflectivity contrast, resonance wavelength and linewidth of the modes are obtained by fitting the experimental reflectivity of the elliptical micropillar with the Lorentzian.

The different optical elements are described by the following Jones matrices:

Linear *H* polarizer	$P_H = \begin{bmatrix} 1 & 0 \\ 0 & 0 \end{bmatrix}$
Linear *V* polarizer	$P_V = \begin{bmatrix} 0 & 0 \\ 0 & 1 \end{bmatrix}$
Quarter waveplate	$W_{\lambda/4} = e^{-i\pi/4} \begin{bmatrix} 1 & 0 \\ 0 & -i \end{bmatrix}$
Half waveplate	$W_{\lambda/2} = \begin{bmatrix} 1 & 0 \\ 0 & -1 \end{bmatrix}$

Table S1: Jones matrices of waveplates and polarizers in the *H/V* basis [1,2]

The polarization state of the reflected beam is then calculated as:

$$|\psi_{refl}\rangle = M_{sample} \times M(\theta_2) W_{\lambda/2} M(\theta_2)^{-1} \times M(\theta_1) W_{\lambda/4} M(\theta_1)^{-1} \times |\psi_{in}\rangle.$$

(1) Goldstein, D. H. *Polarized Light*, 3rd Edition.; CRC Press, 2011. https://doi.org/10.1201/b10436.
(2) Teich, M. C.; Saleh, B. E. A. Fundamentals of Photonics.

III. Optical and acoustic fields

In Fig. S1, we include the simulated acoustic and optical fields as a function of the position in a planar microcavity structure using the transfer matrix method. This layer structure corresponds to the one shown in Fig. 1a of the main text. Note that the optical and acoustic fields present exactly the same spatial profile. The same profile will be found in an elliptical micropillar in the vertical direction. We also include in Fig. S2 the optical mode splitting measured as a function of the micropillar elipticity, which constitutes the main feature to control the polarization rotation.

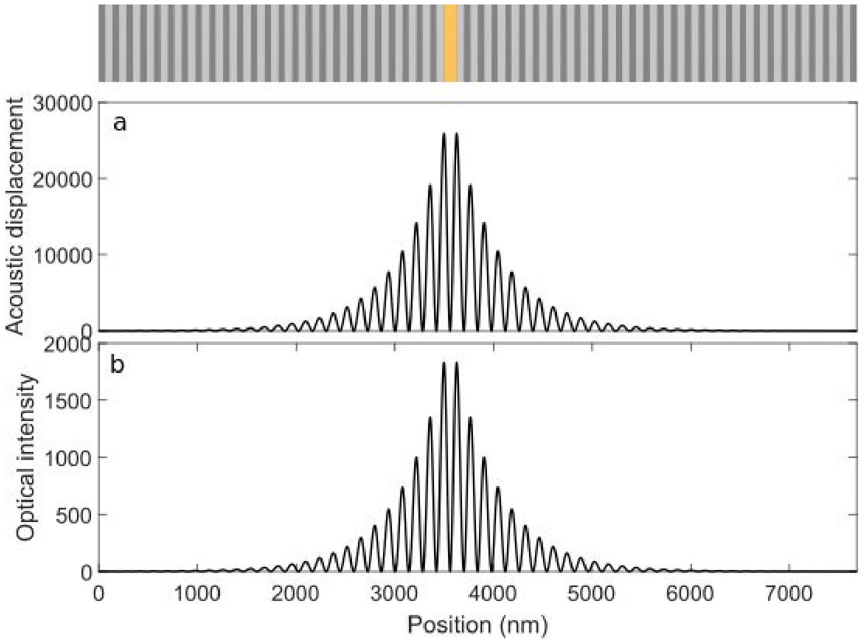

Fig S1: Simulated fields as a function of the position in the structure using the transfer matrix method. The structure is considered embedded in between air and a GaAs substrate. (a) Acoustic displacement of the confined mode and (b) optical field of the confined mode.

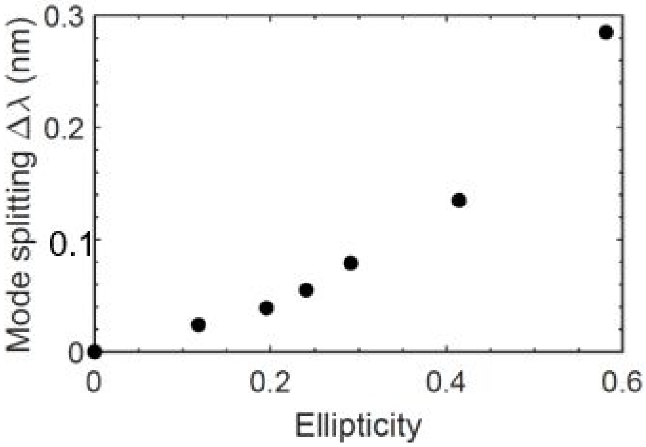

Fig. S2: Optical mode splitting measured as a function of the micropillar elipticity
